# Performance Characteristics of the NeuroEXPLORER, a Next-Generation Human Brain PET/CT Imager

**DOI:** 10.2967/jnumed.124.267767

**Published:** 2024-08

**Authors:** Hongdi Li, Ramsey D. Badawi, Simon R. Cherry, Kathryn Fontaine, Liuchun He, Shannan Henry, Ansel T. Hillmer, Lingzhi Hu, Nikkita Khattar, Edwin K. Leung, Tiantian Li, Yusheng Li, Chi Liu, Peng Liu, Zhenrui Lu, Stanislaw Majewski, David Matuskey, Evan D. Morris, Tim Mulnix, Negar Omidvari, Suranjana Samanta, Aaron Selfridge, Xishan Sun, Takuya Toyonaga, Tommaso Volpi, Tianyi Zeng, Terry Jones, Jinyi Qi, Richard E. Carson

**Affiliations:** 1 United Imaging Healthcare North America, Houston, Texas;; 2 University of California, Davis, Davis, California;; 3 Yale University, New Haven, Connecticut; and; 4 United Imaging Healthcare, Shanghai, China

**Keywords:** brain PET, NEMA, NeuroEXPLORER, DOI, high resolution

## Abstract

The collaboration of Yale, the University of California, Davis, and United Imaging Healthcare has successfully developed the NeuroEXPLORER, a dedicated human brain PET imager with high spatial resolution, high sensitivity, and a built-in 3-dimensional camera for markerless continuous motion tracking. It has high depth-of-interaction and time-of-flight resolutions, along with a 52.4-cm transverse field of view (FOV) and an extended axial FOV (49.5 cm) to enhance sensitivity. Here, we present the physical characterization, performance evaluation, and first human images of the NeuroEXPLORER. **Methods:** Measurements of spatial resolution, sensitivity, count rate performance, energy and timing resolution, and image quality were performed adhering to the National Electrical Manufacturers Association (NEMA) NU 2-2018 standard. The system’s performance was demonstrated through imaging studies of the Hoffman 3-dimensional brain phantom and the mini-Derenzo phantom. Initial ^18^F-FDG images from a healthy volunteer are presented. **Results:** With filtered backprojection reconstruction, the radial and tangential spatial resolutions (full width at half maximum) averaged 1.64, 2.06, and 2.51 mm, with axial resolutions of 2.73, 2.89, and 2.93 mm for radial offsets of 1, 10, and 20 cm, respectively. The average time-of-flight resolution was 236 ps, and the energy resolution was 10.5%. NEMA sensitivities were 46.0 and 47.6 kcps/MBq at the center and 10-cm offset, respectively. A sensitivity of 11.8% was achieved at the FOV center. The peak noise-equivalent count rate was 1.31 Mcps at 58.0 kBq/mL, and the scatter fraction at 5.3 kBq/mL was 36.5%. The maximum count rate error at the peak noise-equivalent count rate was less than 5%. At 3 iterations, the NEMA image-quality contrast recovery coefficients varied from 74.5% (10-mm sphere) to 92.6% (37-mm sphere), and background variability ranged from 3.1% to 1.4% at a contrast of 4.0:1. An example human brain ^18^F-FDG image exhibited very high resolution, capturing intricate details in the cortex and subcortical structures. **Conclusion:** The NeuroEXPLORER offers high sensitivity and high spatial resolution. With its long axial length, it also enables high-quality spinal cord imaging and image-derived input functions from the carotid arteries. These performance enhancements will substantially broaden the range of human brain PET paradigms, protocols, and thereby clinical research applications.

## Visual Abstract

**Figure:**
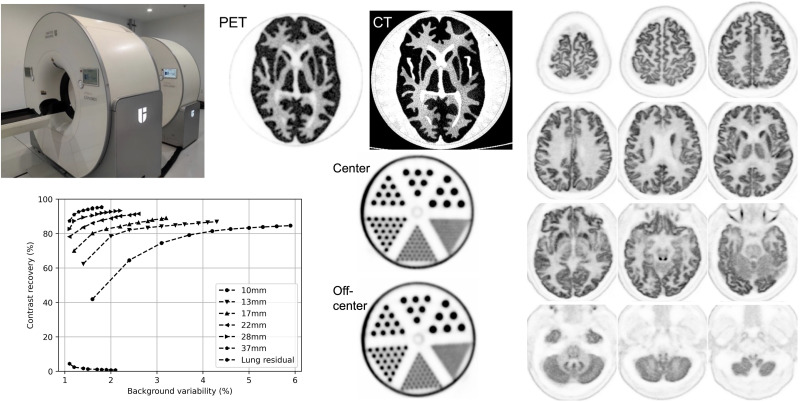

The use of brain PET in research has spanned more than 5 decades. PET has been shown to be a valuable tool for assessing changes in flow, metabolism, and receptor occupancy resulting from pharmacologic or cognitive stimulation. This accomplishment is made possible through the integration of PET imagers, quantitative reconstruction techniques, kinetic modeling, and targeted radiotracers, collectively enabling a broad range of quantitative approaches for studying pharmacologic and physiologic aspects of the brain (1). This powerful modality can also address basic neuroscience questions in humans and assess the brain pharmacokinetics of novel drugs. Subsequently, the same tools are applied clinically, for example, to diagnose and stage neurodegenerative conditions such as Parkinson and Alzheimer diseases (2*,*^3^) and directly monitor the effects of drugs (4).

Nonetheless, dynamic assessment of neurochemical-specific brain function remains challenging. Accurate quantification demands high-sensitivity imaging across the entire brain, to define and quantify important areas such as subnuclei within the thalamus and amygdala, hippocampal subfields, and midbrain nuclei (e.g., superior and inferior colliculi). Achieving ultrahigh resolution is also crucial to enhance the quantification of small brain structures by minimizing partial-volume effects (5).

For kinetic modeling, having precise measurements of the arterial input function is crucial for accurate quantification. High-sensitivity and high-resolution imaging offers the potential for obtaining image-derived input functions from the carotid arteries, reducing or eliminating the necessity for arterial sampling (6).

Existing state-of-the-art commercial PET imaging systems, including the 25-y-old High Resolution Research Tomograph (Siemens) (7), do not adequately meet this challenge. For the High Resolution Research Tomograph, the limited sensitivity and lack of time-of-flight (TOF) capability produce insufficient statistical precision for measurements, especially in small regions. In the last decade, there has been renewed interest in dedicated brain PET systems (8). Systems developed in both commercial and academic settings are demonstrating substantially improved performance compared with clinical whole-body PET systems (9–17). Despite this progress, these systems must demonstrate whether they meet the goals of comprehensively capturing the wide dynamic range of brain function with both high temporal and high spatial precision.

In 2019, the EXPLORER consortium developed a total-body PET scanner with a long axial field of view (AFOV) of almost 2 m, boosting sensitivity by up to 40 times (18*,*^19^). Studies on this system clearly demonstrated that high sensitivity plays a pivotal role in obtaining low-noise, high-resolution images at the temporal resolution desired for dynamic imaging and kinetic modeling (20*,*^21^).

Subsequently, the NeuroEXPLORER (NX) was developed collaboratively by Yale, the University of California, Davis, and United Imaging. The NX incorporates a long-AFOV concept with a 49.5-cm axial length and innovative high-resolution PET detectors featuring TOF and depth-of-interaction (DOI) encoding technologies to yield high sensitivity and high spatial resolution across the entire head. Including the impact of TOF, the NX was designed to produce an order-of-magnitude higher effective sensitivity than is possible with the High Resolution Research Tomograph to yield significantly improved image quality. The objective of this study was to evaluate the NX system’s physical performance and quantitative accuracy following the National Electrical Manufacturers Association (NEMA) NU 2-2018 standard and to provide an initial demonstration of the system’s capabilities with phantom and human images.

## MATERIALS AND METHODS

### System Parameters

**FIGURE 1.**
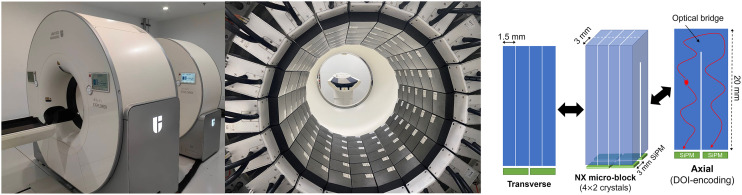
NX system. (Left) Overview of PET/CT with CT in front. (Center) NX PET detector ring (before removal of shoulder cutouts). (Right) U-shaped light-sharing detector array design providing DOI information.

The NX combines an 80-slice CT scanner (United Imaging uCT 550) with a dedicated brain PET scanner as shown in Figure 1. The CT scanner, capable of performing helical acquisitions with a minimum slice thickness of 0.5 mm and a minimum rotation time of 0.5 s, can be used in a low-dose mode for PET attenuation correction. There is an 80-cm gap between the CT and PET components, streamlining patient preparation, tracer injection, blood sampling, and other clinical procedures that require easy access to the subject.

The NX PET consists of 20 detector modules (each containing 5 or 6 blocks aligned axially) forming a cylindric detector ring with a diameter and AFOV of 52.4 and 49.5 cm, respectively. A smaller ring diameter was not used because of patient comfort concerns, especially for long dynamic scans. In addition, the larger bore may facilitate pediatric imaging. To minimize parallax errors in a compact detector ring, a depth-encoding detector design is necessary. The NX DOI detectors (Fig. 1) incorporate a U-shaped light-sharing detector array design with single-ended readout by silicon photomultipliers (SiPMs). Every U-shaped element comprises 2 lutetium-yttrium oxyorthosilicate crystals measuring 1.56 × 3.07 × 20.0 mm, and a microblock is created by combining 4 U-shaped elements. This microblock is then read out by a 2 × 2 array of 3-mm SiPMs. DOI is read out in 8 bins, determined by the proportion of light detected by the 2 axial SiPMs (Fig. 1). A DOI resolution of less than 4 mm was determined on the benchtop from coincidence data acquired while a point source was stepped along the depth direction of the detector (22). Overall, the detector delivers high-spatial and -energy resolution and very good TOF capability. Using individual-event DOI and energy information, we detect and recover intercrystal scatter events that occur between adjacent microblocks (23).

An NX detector block consists of 12 × 12 microblocks. In total, the NX has 131,328 crystals read out by 65,664 SiPMs organized into 114 blocks. To enhance signal quality and reduce dead-time effects, we used a custom-designed 32-channel application-specific integrated circuit capable of precise time and amplitude measurements. The front-end electronics, consisting of 2,052 application-specific integrated circuits and 248 field-programmable gate arrays, handle real-time event localization and intercrystal scatter correction while maintaining low dead time.

To optimize detection sensitivity, we created a partial sixth ring by eliminating 3 blocks on both sides of the ring. This adaptation allows for different shoulder sizes, maintaining the brain at the scanner’s central position for optimal sensitivity in the primary region of interest. It also improves sensitivity for imaging the carotid arteries. Furthermore, an integrated markerless 3-dimensional camera allows real-time motion tracking during imaging (24).

The console reconstruction is a 3-dimensional ordered-subset expectation-maximization (OSEM) algorithm including TOF, DOI, and point spread function (PSF) modeling. Data from the full axial acceptance angle are included. The console reconstruction incorporates DOI rebinning, which uses the DOI data to reassign the endpoints of the lines of response (LORs) to the front surface of appropriate nearby detectors. This facilitates rapid reconstruction and ensures smooth compatibility with established commercial reconstruction software (25).

### NEMA NU 2-2018 Performance Evaluation

#### Spatial Resolution

We measured spatial resolution at 6 different locations using a 0.25-mm ^22^Na point source (370 kBq, 10 μCi) embedded in a solid disk. The source was positioned at 2 axial positions: the axial center of the system (½ AFOV) and about 6 cm from the end of the tomograph (⅛ AFOV). Within each transaxial plane, resolution measurements were performed at 1-, 10-, and 20-cm radial offsets. The list-mode data (energy window of 430–800 keV) containing LORs with oblique angles less than 3.7° were adjusted by DOI rebinning and transformed into 2-dimensional sinograms using Fourier rebinning (26). The data were then reconstructed using a 2-dimensional filtered backprojection algorithm without attenuation correction, scatter correction, or postsmoothing, with a voxel size of 0.5 × 0.5 × 0.5 mm; the TOF information was not used. The axial, radial, and tangential resolutions were evaluated according to the NEMA NU 2-2018 standard.

#### Sensitivity

The sensitivity measurement was performed using a NEMA PET sensitivity phantom consisting of 5 concentric aluminum sleeves, each 70 cm in length. A 70-cm-long polyethylene tube filled with a solution of 13 MBq (0.35 mCi) of ^18^F was inserted into the aluminum sleeves and positioned at the center of the transaxial field of view (FOV). Five 300-s acquisitions were conducted, successively removing the outermost sleeve to extrapolate attenuation-free sensitivity. The collected list-mode data were binned into sinograms using single-slice rebinning to estimate the total sensitivity and axial slice sensitivity. The entire process was replicated with the line source positioned 10 cm off-center within the transaxial FOV.

#### Count Rate Performance

Following the NEMA NU 2-2018 standard, count rate performance was evaluated using a scatter phantom consisting of a 70-cm-long polyethylene cylinder with a 20-cm diameter. The line source was initially loaded with 1.54 GBq (41.6 mCi) of ^18^F-FDG. In total, 34 acquisition frames were acquired with increasing durations as the activity decayed. Random estimation was obtained from the delayed coincidence channel. The scatter fraction was determined in accordance with the NEMA standard, and Monte Carlo scatter simulation was used in the image reconstruction for evaluation of accuracy (relative count error). Dead-time correction was based on a nonparalyzable model. System count rates, including trues, randoms, scatters, total counts, ideal trues, and the noise-equivalent count rate, were plotted alongside their corresponding activity concentrations. Additionally, a plot illustrating the scatter fraction in relation to activity concentration was generated. The ideal trues count rate was estimated from a linear fit to the trues rate at low activities when dead-time effects are negligible; count loss due to dead time was calculated by comparing measured and ideal trues count rates.

#### Energy and Timing Resolution

The data obtained during the count rate performance measurement were used to assess the system timing resolution and energy resolution (27). For source position determination, the acquisition data, at an activity concentration just below the peak noise-equivalent count rate, were reconstructed using non-TOF OSEM with all corrections applied. Only coincidences occurring within the ±20-mm region of interest centered on the line source were used to obtain the timing histogram. For each coincidence dataset, the time error was computed by measuring the difference between the actual TOF data and its expected TOF offset based on the point closest to the line source on the corresponding LOR. Scatter and random coincidences were subtracted using the tails of the TOF histograms. For energy resolution, crystal-level energy spectra with a 1-keV energy bin width were combined into a single spectrum. The full width at half maximum of energy resolution was determined by the linear interpolation between the adjacent pixels at half the maximum value. This value was determined over all count rates.

#### Image Quality and Accuracy of Corrections

A NEMA NU 2 image-quality phantom was used to evaluate image quality. At the start of image acquisition, the background activity concentration of ^18^F-FDG was 5.3 kBq/mL. Six spheres with diameters of 10, 13, 17, 22, 28, and 37 mm were filled with the ^18^F-FDG solution with an activity concentration ratio of 4.0:1 relative to the background. An adjacent scatter phantom (used for count rate performance measurement) was placed axially to the NEMA image-quality phantom; the line source activity in the scatter phantom was 116 MBq (3.14 mCi), the duration of the scan was 15 min, and 3 sequential measurements were acquired. Images were reconstructed using the standard TOF-DOI-PSF OSEM algorithm with all corrections applied. Mean, minimum, and maximum results are shown at multiple iterations with 10 subsets (voxel size, 1.8 × 1.8 × 1.72 mm).

### Phantom Studies

A 3-dimensional Hoffman brain phantom was filled with 30 MBq (0.81 mCi) of ^18^F-FDG, placed at the center of the FOV, and scanned for 30 min. A mini-Derenzo resolution phantom was used to further evaluate the PET resolution performance. This phantom contained rods of various diameters (1.2, 1.6, 2.4, 3.2, 4.0, and 4.8 mm), and the center spacing between rods was twice the rod diameter. The phantom was filled with ^18^F-FDG and imaged for 10 min at the center of the FOV (36.8 MBq, 0.99 mCi) and for 15 min at 85 mm off-center (26.5 MBq, 0.72 mCi), with the rod axes perpendicular to the transaxial plane. Both phantoms underwent image reconstruction using the console TOF-DOI-PSF OSEM algorithm with 7 iterations and 10 subsets. No postreconstruction filtering was applied, and the image voxel size was 0.4 × 0.4 × 0.6 mm. For the mini-Derenzo phantom, 2 additional reconstructions were performed with offline list-mode algorithms: one using no DOI information, and the other directly using DOI coordinates, that is, no DOI rebinning.

### Human Study

A 58-y-old healthy male volunteer participated in a brain ^18^F-FDG PET protocol approved by the Yale University Human Investigation Committee and the Yale Radiation Safety Committee. Written informed consent was obtained. The subject was administered 327 MBq (8.8 mCi) in a bolus–plus–continuous-infusion protocol. A CT scan was acquired for attenuation correction. Dynamic data were acquired in list mode for 90 min. Reconstructed images contained 600 × 600 × 990 voxels with a voxel size of 0.5 × 0.5 × 0.5 mm. TOF-DOI-PSF OSEM was used with 7 iterations and 10 subsets and all corrections. A static ^18^F-FDG PET image was generated by summing over 40- to 70-min data and registered to T1-weighted MR images using FSL (28) for anatomic localization. This choice of period was based on the camera data, which showed minimal subject motion during that time.

## RESULTS

### NEMA NU 2-2018 Performance Evaluation

#### Spatial Resolution

The transverse (averaged radial and tangential) spatial resolution using filtered backprojection was 1.64, 2.06, and 2.51 mm, and axial resolution was 2.73, 2.89, and 2.93 mm in full width at half maximum, with radial offsets of 1, 10, and 20 cm from the center, respectively (Table 1).

**TABLE 1.** Spatial Resolution of ^22^Na Point Source Measured with Filtered Backprojection

Axial position	Radial off-center position (cm)	Full width at half maximum (mm)		
		Radial	Tangential	Axial
½ AFOV	1	1.63	1.68	2.55
	10	2.10	2.06	2.97
	20	2.17	2.87	2.82
⅛ AFOV	1	1.76	1.47	2.91
	10	1.99	2.08	2.80
	20	2.20	2.78	3.05

#### Sensitivity

**FIGURE 2.**
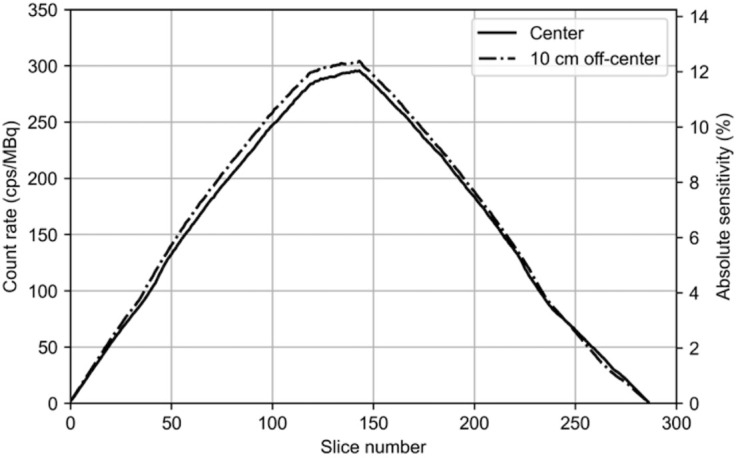
Slice sensitivity profiles along 49.5-cm AFOV measured with 70-cm line source positioned at transaxial center and 10 cm off-center. Data are shown for 287 slices calculated by single-slice rebinning. Count rates and absolute sensitivities are displayed on left and right vertical axes, respectively. Slice sensitivity was determined by normalizing slice count rate data from entire phantom by ratio of phantom length to slice thickness (1.725 mm).

The NEMA total-system sensitivities were 46.0 and 47.6 kcps/MBq at the center and at the 10-cm radial offset, respectively (Fig. 2). An absolute sensitivity of 11.8% was achieved at the center of the FOV.

#### Count Rate Performance

**FIGURE 3.**
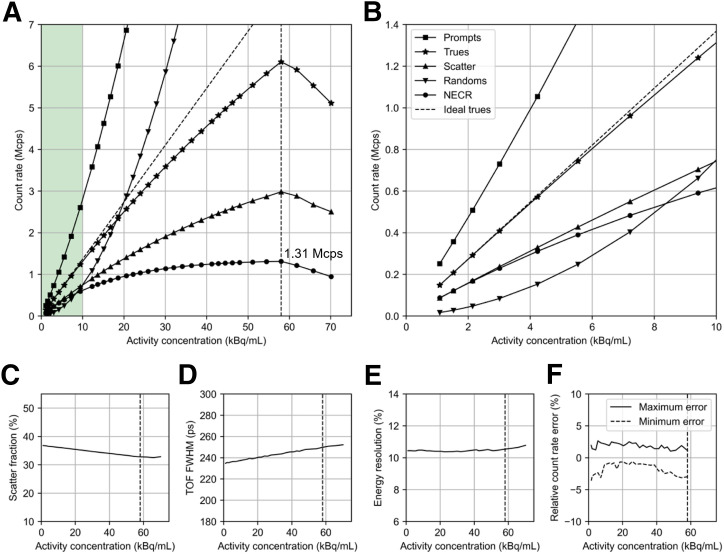
Count-rate performance of NX. (A) Prompts, trues, scatter, randoms, and noise-equivalent count rate. (B) Zoomed display of A at ∼0–10 kBq/mL, which corresponds to typical activity level for human brain imaging. (C) Scatter fraction. (D) TOF resolution. (E) Energy resolution. (F) Relative count rate error as function of activity concentration. Vertical dashed line denotes noise-equivalent count rate peak position at 58.0 kBq/mL, corresponding to 1,275 MBq (34.5 mCi). NECR = noise-equivalent count rate.

Figure 3 shows the count rate performance data for the NX. The peak noise-equivalent count rate was 1.31 Mcps at an activity concentration of 58.0 kBq/mL. Above this point, the count rate drops because of bandwidth limitations of the data acquisition hardware; note that this activity level is well above that found in brain imaging. The average scatter fraction was 36.5% at 5.3 kBq/mL (Fig. 3). Count losses of 5.1% and 10.2% were noted at 12.3 and 23.9 kBq/mL, respectively, because of system dead time. The maximum count rate error was less than 5.0%.

#### Energy and Timing Resolution

The measured average system TOF resolution was 236 ps, and the energy resolution was 10.5% (full width at half maximum) at 5.3 kBq/mL. Both values were stable with count rate, with variations of 2.1% and 0.8% for TOF and energy resolution, respectively.

#### Image Quality and Accuracy of Correction

**FIGURE 4.**
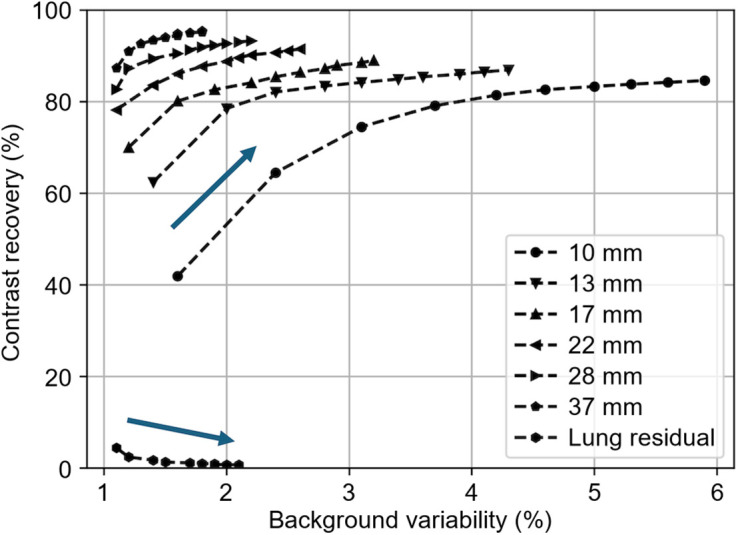
Contrast recovery coefficient measurements derived from image-quality phantom. OSEM results are shown for 1–10 iterations with 10 subsets; arrow denotes increasing iterations. Values are mean results from three 15-min acquisitions.

Table 2 summarizes the contrast recovery coefficient, background variability, and residual error in the cold lung region for the 4.0:1 sphere-to-background ratio, based on 3 combined measurements of the image-quality phantom. The average contrast recovery coefficient varied from 74.5% (10-mm sphere) to 92.6% (37-mm sphere), and background variability varied from 3.1% to 1.4% at 3 iterations (10 subsets). Plotting the contrast recovery coefficient versus background variability for iterations 1–10 suggests that the contrast recovery coefficients reach convergence in most spheres at 8 iterations (Fig. 4).

**TABLE 2.** Measured Contrast Recovery Coefficient and Background Variability for 3 and 7 Iterations of OSEM Reconstruction

	Sphere size						
Parameter	10 mm	13 mm	17 mm	22 mm	28 mm	37 mm	Lung residual error
Contrast recovery coefficient							
3-iteration OSEM							
Mean	74.5	82.1	82.6	86.1	89.4	92.6	1.7
Maximum	76.1	84.3	84.7	87.0	89.8	93.1	1.7
Minimum	72.6	80.3	81.5	85.0	88.9	91.6	1.7
7-iteration OSEM							
Mean	83.3	85.4	87.2	90.2	92.3	94.8	0.9
Maximum	85.6	87.9	90.3	91.7	92.9	95.5	0.9
Minimum	80.9	82.2	84.9	88.2	91.6	93.4	0.9
Background variability							
3-iteration OSEM							
Mean	3.1	2.4	1.9	1.6	1.4	1.3	1.4
Maximum	3.4	2.6	2.0	1.7	1.5	1.3	1.4
Minimum	2.6	2.1	1.8	1.5	1.4	1.3	1.4
7-iteration OSEM							
Mean	5.0	3.6	2.8	2.2	1.9	1.6	1.9
Maximum	5.4	3.9	3.0	2.4	2.0	1.7	1.9
Minimum	4.2	3.1	2.5	2.1	1.8	1.5	1.8

Results are reported as mean, minimum, and maximum percentages over 3 measurements.

### Phantom Studies

**FIGURE 5.**
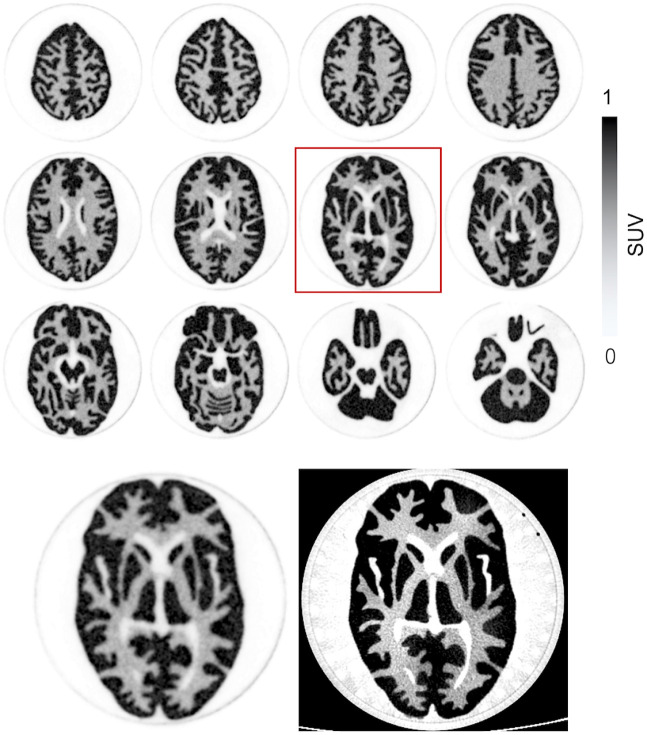
(Top) Reconstructed PET images of Hoffman 3-dimensional brain phantom. PET images were created with console reconstruction algorithm using 7 iterations and 10 subsets with voxel size of 0.4 ×0.4 × 0.6 mm. (Bottom) Matched slices of PET (highlighted slice from top) compared with CT image. PET images are displayed on SUV scale.

Twelve transverse slices of the Hoffman phantom are shown in Figure 5. There is clear delineation of the fine structures in the phantom. This is most clearly portrayed when a central PET slice is compared with the matching CT slice, which depicts the accuracy of the border between gray and white matter structures.

**FIGURE 6.**
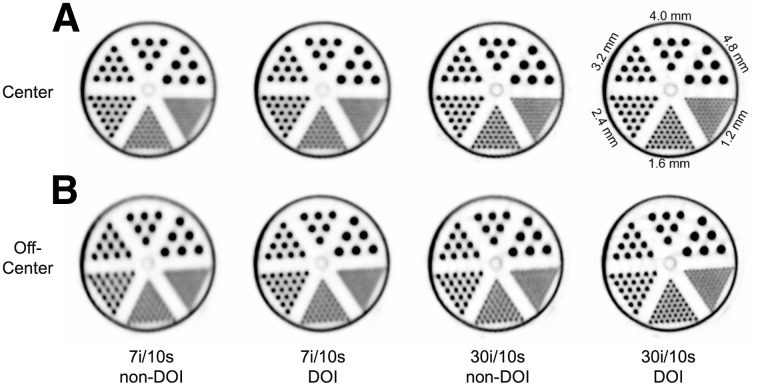
Image slices reconstructed from mini-Derenzo phantom, positioned at center (A) and 85 mm off-center (B) of transaxial plane, using TOF-PSF OSEM algorithm with voxel size of 0.4 × 0.4 × 0.6 mm. (Column 1) Seven iterations and 10 subsets (7i/10 s) with non-DOI offline reconstruction. (Column 2) Seven iterations and 10 subsets using DOI rebinning in console reconstruction. (Column 3) Thirty iterations and 10 subsets (30i/10 s) with non-DOI offline reconstruction. (Column 4) Thirty iterations and 10 subsets with full DOI offline list-mode reconstruction.

Figure 6 shows the results of mini-Derenzo phantom images and the impact of DOI. The effects of uncorrected DOI are clearly seen by comparison of the central and offset images without DOI correction. Using the console reconstruction with DOI rebinning, PSF, and TOF, the 1.6-mm hot rods can be resolved using 7 or more iterations for both center and off-center cases. Comparing columns 1 and 2, the addition of DOI information produces the most notable improvement for the off-center phantom. To further assess DOI effects, list-mode reconstructions with 30 iterations were performed without DOI and with full DOI information. Increasing iterations further improved the images, and with DOI, the 1.2-mm rods were more clearly resolved. Overall, reconstructions using DOI information showed advantages at both a low and a high number of iterations compared with non-DOI results.

### Human Study

**FIGURE 7.**
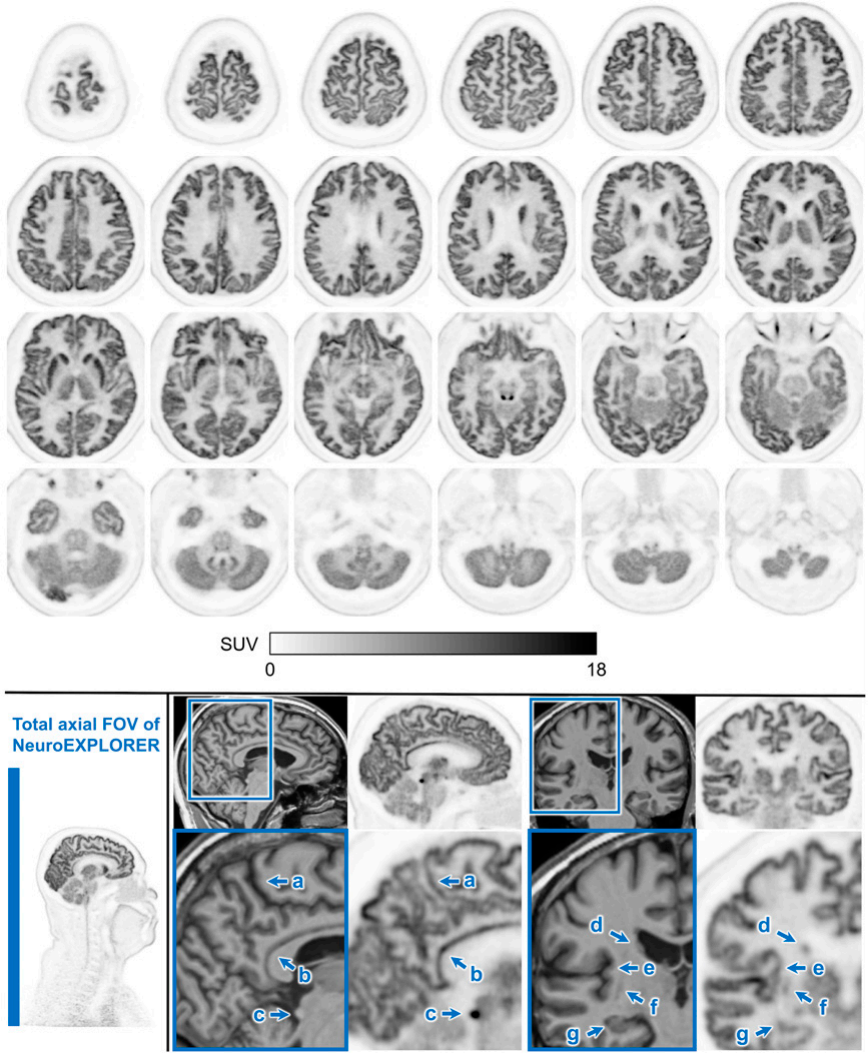
^18^F-FDG PET image (40–70 min after injection) on NX. (Top) Top-to-bottom axial slices showing excellent spatial resolution of NX, which provides clear delineation of cortical ribbon along with subcortical nuclei. (Bottom, far left) Sagittal image showing long AFOV of NX (49.5 cm), which allows inclusion of neck and upper chest structures. (Bottom right) Two pairs of T1-weighted MRI (left) and NX (right) image slices. Beneath are zoomed-in regions demonstrating NX’s high spatial resolution for cortical ribbon structures and small brain nuclei, including marginal sulcus (a), posterior cingulate cortex (b), inferior colliculus (c), caudate (d), insular cortex (e), putamen (f), and hippocampus (g).

Reconstructed human NX images are shown in Figure 7, demonstrating very high resolution in the cortex and subcortical structures. The improved visualization is clearly seen in marginal sulcus, posterior cingulate cortex, inferior colliculus, caudate, insular cortex, putamen, and hippocampus. The figure also depicts the full AFOV of the NX system, which provides improved capability for imaging the carotid arteries, head and neck tumors, and cervical spinal cord.

## DISCUSSION

Here, we have presented the performance and first human images of the NX, a next-generation human brain PET/CT system. The design of this system was based on our long experience in brain PET, which showed that high resolution is critically important to measure activity in small brain nuclei and that high sensitivity may play an even greater role to provide sufficient statistics to reconstruct images that fully use the system’s resolution capabilities.

The results for spatial resolution from point sources in air were shown with filtered backprojection. If the TOF-DOI-PSF OSEM reconstruction was applied, the transverse and axial spatial resolutions were substantially improved, with only minor degradation at off-center locations. Specifically, with the addition of a 5% warm background to the point source, the transverse and axial spatial resolutions at the center improved to 1.43 and 1.54 mm, respectively, at 20 iterations (10 subsets). However, we recognize that these OSEM values are likely to be better than what can be achieved in human studies because of the lack of sufficient background activity (29); these results therefore cannot be considered reliable. Nevertheless, considering the clear improvement in phantom image quality provided by OSEM using TOF and DOI (Figs. 5 and 6), it is important to recognize that filtered backprojection results do not provide an accurate measure of real-world system resolution. Ultimately, human brain image quality will depend on the choice of reconstruction parameters, to balance image resolution and voxel noise. Further work is required to characterize the spatial resolution of this system more meaningfully.

For a brain PET scanner with an extended axial length and wide acceptance angles, depth encoding plays a crucial role in reducing parallax errors, to provide nearly uniform spatial resolution across the entire FOV. In addition to precisely defining the LORs, DOI information enhances TOF accuracy by accounting for the variation in the pathlengths of light photons. Ignoring the DOI information resulted in a degradation of TOF resolution of about 99 ps. As demonstrated here, despite having smaller detectors and reduced light collection efficiency, after TOF compensation by the DOI, the NX achieves a TOF resolution of 236 ps, comparable to that of state-of-the-art clinical PET/CT systems with larger detectors.

By discretizing DOI into 8 bins per NX detector, the total number of DOI-based LORs increases 64-fold, which elevates spatial sampling, enhancing axial resolution, particularly for long-AFOV PET with large acceptance angles. This is clearly visualized with the improved image resolution in Figure 6.

Intercrystal scatter events result in inaccurate localization of annihilation photon interactions within detectors. Monte Carlo simulation studies indicate a higher proportion of such scattered events within each LOR within smaller detectors. Integrating DOI information and combining it with event energy can reduce intercrystal scatter events while restoring the original event energy and position information, potentially improving image resolution (23). Currently, NX intercrystal scatter localization is based on higher energy, and optimization using energy and DOI information is under way.

Unlike traditional PET, for which more activity leads to an increased scatter fraction due to event pileup, the NX shows a slight decrease in scatter fraction at high count rates, perhaps because of slightly insufficient cooling under high-count-rate and high-power-consumption conditions; this requires further investigation.

In high-resolution PET scanners, even minor patient movement can negatively impact quantification and produce motion artifacts. The NX is equipped with markerless motion-tracking hardware featuring a real-time stereovision camera with structured light to image the surface of the subject’s head at 10–30 Hz, as well as a real-time data processing unit using an iterative-closest-point 3-dimensional point cloud registration algorithm (24). We are currently evaluating motion correction performance on the NX using the event-based motion correction capabilities of the MOLAR reconstruction platform (30*,*^31^). The vendor-provided console reconstruction does not currently use motion vectors for event-based image reconstruction; instead, frame-based postreconstruction methods are used. The console software also uses a DOI rebinning strategy to efficiently reconstruct all images, albeit at the cost of a slight reduction in image resolution. The incorporation of full list-mode DOI reconstruction is under way.

As demonstrated by the human images, the great sensitivity and spatial resolution of the NX provides a platform for imaging tasks previously considered difficult or impossible, such as accurate imaging of small subcortical regions with focal tracer uptake, low-density binding sites (e.g., cortical dopamine receptors), cervical spinal cord uptake, neurotransmitter dynamics, and carotid arteries for image-derived input functions.

## CONCLUSION

The NX achieves very high spatial resolution and sensitivity, as shown by the NEMA NU-2 measurements and the images presented here. This performance is made possible by U-shaped DOI lutetium-yttrium oxyorthosilicate detectors combined with a single-ended SiPM-based application-specific integrated circuit readout. The system delivers a timing resolution of 236 ps. In view of these design and performance features, the NX introduces exciting possibilities for human brain PET protocols and clinical research applications, pushing the boundaries of what can be achieved in neuroimaging.

## DISCLOSURE

This work was supported by NIH BRAIN Initiative grant U01EB029811. The University of California, Davis, has a research agreement and a sales-based revenue-sharing agreement with United Imaging Healthcare. No other potential conflict of interest relevant to this article was reported.

## Notes

KEY POINTS **QUESTION:** What is the performance of the next-generation dedicated brain PET imager—the NX—according to the NEMA NU 2-2018 standard? **PERTINENT FINDINGS:** The NX provides a spatial resolution below 2 mm, a sensitivity of 46 cps/kBq, and a timing resolution of 236 ps and includes a built-in camera for real-time motion correction. **IMPLICATIONS FOR PATIENT CARE:** The NX allows for operation at high resolutions or low doses, with the potential to substantially improve quantification and to allow for early detection and treatment monitoring of neurodegenerative and neuropsychiatric disorders.
